# Therapeutic Effect of Bee Venom and Melittin on Skin Infection Caused by *Streptococcus pyogenes*

**DOI:** 10.3390/toxins14100663

**Published:** 2022-09-23

**Authors:** Seongjae Bae, Hyemin Gu, Mi-Gyeong Gwon, Hyun-Jin An, Sang-Mi Han, Sun-Jae Lee, Jaechan Leem, Kwan-Kyu Park

**Affiliations:** 1 Department of Pathology, School of Medicine, Catholic University of Daegu, Gyeongsan 42472, Korea; 2 Department of Agricultural Biology, National Academy of Agricultural Science, RDA, Wanju 54875, Korea; 3 Department of Immunology, School of Medicine, Catholic University of Daegu, Gyeongsan 42472, Korea

**Keywords:** bee venom, melittin, skin infection, *Streptococcus pyogenes*

## Abstract

*Streptococcus pyogenes* (*S. pyogenes*) bacteria cause almost all primary skin infections in humans. Bee venom (BV) and melittin (Mel) have multiple effects, including antibacterial and anti-inflammatory activities. This study aims to demonstrate their effects on bacterial mouse skin infection using *S. pyogenes*. The dorsal skin was tape-stripped, then *S. pyogenes* was topically applied. BV or Mel were topically applied to the lesion. The tissues were stained with hematoxylin and eosin, while immunohistochemical staining was performed with anti-neutrophil. *S. pyogenes*-infected skin revealed increased epidermal and dermal layers, but it was reduced in the BV and Mel groups. Finding increased neutrophils in the mice infected with *S. pyogenes*, but the BV and Mel mice showed decreased expression. These results suggest that BV and Mel treatments could reduce the inflammatory reactions and help improve lesions induced by *S. pyogenes* skin infection. This study provides additional assessment of the potential therapeutic effects of BV and Mel in managing skin infection caused by *S. pyogenes*, further suggesting that it could be a candidate for developing novel treatment alternative for streptococcal skin infections.

## 1. Introduction

As the largest organ in the human body, the skin is colonized by many beneficial microorganisms. It serves as a physical barrier preventing the invasion of foreign pathogens while also providing a home to these commensal microbiota [1]. In circumstances where the barrier is broken or the balance between commensals and pathogens is disturbed, skin or even systemic disease can result [1]. In particular, skin infections are frequently found in community and hospital settings, and these can be caused by bacteria, fungi, viruses, and parasites [2].

*Streptococcus pyogenes* (*S. pyogenes*) and *Staphylococcus aureus* (*S. aureus*) bacteria cause almost all primary skin infections in humans [3]. *S. pyogenes*, or group A *streptococcus*, is a Gram-positive bacterium that expresses the Lancefield group A carbohydrate [4], leading to diverse clinical manifestations ranging from superficial epithelial to extremely invasive infections [5]. *S. pyogenes* also express a variety of virulence factors that are crucial for adherence, colonization, dissemination of infection and immune evasion [6]. The various toxic factors are expressed with different functions depending on infection site and stage [5]. *S. pyogenes* infections are commonly associated with an intense inflammatory state, and exhibit an increased vascular permeability and recruitment of neutrophils to the site of infection [7]. Certain *S. pyogenes* strains acquire access to the skin through abrasions and skin lesions [8], with epidermal infections including impetigo and ecthyma and dermal infections such as erysipelas, cellulitis, and necrotizing fasciitis [9].

Current treatment of *S. pyogenes* infections is based on conventional antibiotics and symptom management [9], with penicillin and clindamycin being the main antibacterial treatments for streptococcal skin infections [10]. Although antibiotics such as penicillin have long been effective in treating *S. pyogenes*, there are reports of several issues associated with this therapy, including a weakening of the antibiotic effect [11]. Moreover, the side effects of penicillin in terms of allergic reaction and high immunogenicity cannot be ignored [12]. Clindamycin-resistant *S. pyogenes* have also been reported with increasing frequency throughout the world [13]. As this bacteria steadily evolves to develop resistance against various antibiotics, it is necessary to establish new treatments or different strategies to promote the careful use of existing drugs. Exploring effective alternative substances without risk of resistance and with fewer side effects for application in the treatments of *S. pyogenes* skin infections are consequently of great importance.

Among the various products of natural origin, bee venom (BV) is particularly rich in bioactive compounds and supports a variety of activities for various causes of disease [14,15]. BV contains a number of bioactive proteins, including melittin (Mel), apamin, adolapin, phospholipases A2 and B, hyaluronidase, serotonin, histamine, dopamine, and noradrenaline [16], and it has traditionally been used to treat many conditions, including arthritis, rheumatism, cancerous tumors, skin diseases, and multiple sclerosis [17]. BV has proven to be an active anti-inflammatory [18] and antibacterial agent against several Gram-positive/negative bacteria strains [14].

Mel is a principal component of BV, accounting for between 40 and 60% of its composition [19]. Mel contains mast cell degranulating peptide, secapin, adolapin, tertiapin, apamin, and enzymes [20]. Many studies have demonstrated Mel’s potential for treating various cancer types and broad spectrum microorganisms [21,22]. Its anti-inflammatory and antimicrobial activities are well-known and have been exploited in complementary anti-bacterial agents [23,24].

Although BV and Mel have been found to inhibit the expression of pro-inflammatory cytokines and to exert anti-inflammatory activity in various skin diseases [25,26], their therapeutic effects on inflammatory skin diseases caused by *S. pyogenes* have not been sufficiently studied. Therefore, this study aims to investigate these effects.

## 2. Results

### 2.1. Effects of BV and Mel Application on Superficial Skin Lesions Caused by S. pyogenes Infection

**Figure 1:**
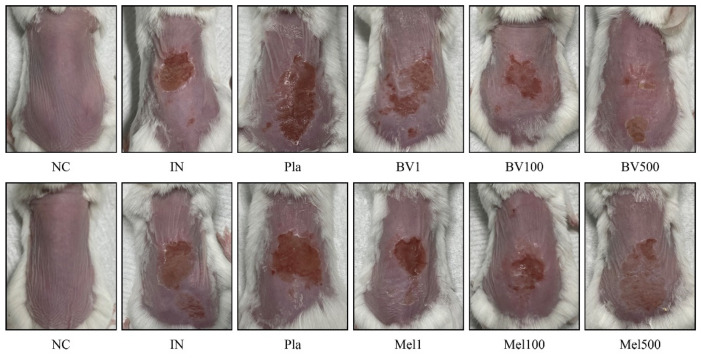
Skin lesions after *S. pyogenes* infection. Mice were superficially infected with *S. pyogenes* (1 × 10^8^ CFU/50 μL) and subsequently treated with BV or Mel, with the first application 48 h after infection and the second and final application 6 h later. At 18 h after the last treatment, the representative images of the back were photographed. Lesions appeared on the skin of all infected mice with a vesicle on an easily ruptured erythematous base, resulting in superficial ulceration covered with purulent discharge that then dried as an adherent yellow crust. Negative control (NC); infected with *S. pyogenes* (IN); vaseline treatment as placebo after infection (Pla); *S. pyogenes* infection with 1, 100, and 500 μg/mL BV cream (BV1, BV100, and BV500); *S. pyogenes* infection with 1, 100, and 500 μg/mL Mel cream (Mel1, Mel100, and Mel500).

**Figure 2:**
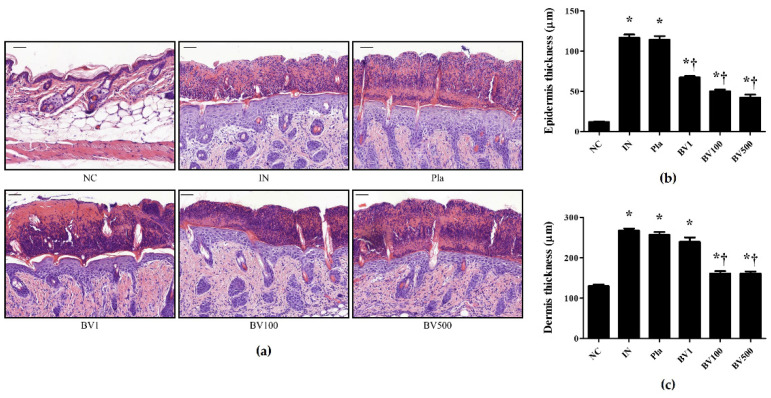
Effect of BV in *S. pyogenes* skin infection models. At 18 h after the last topical application, biopsy specimens were taken and immediately fixed in 10% formalin, embedded in paraffin, and then stained with hematoxylin and eosin (H&E). (**a**) An increase in skin thickness and epidermal hyperplasia surrounding the infection site of *S. pyogenes* was observed by H&E staining. (**b**) Numerical value of epidermis thickness. (**c**) Numerical value of dermis thickness. The graphs are the statistical analysis for each staining. The results are expressed as mean ± SEM of three independent determinations. * *p* < 0.05 compared with the NC group; † *p* < 0.05 compared with the IN group. Magnification ×200, scale bar 50 μm. Negative control (NC); infected with *S.* *pyogenes* (IN); vaseline treatment as placebo after infection (Pla); *S. pyogenes* infection with 1, 100, and 500 μg/mL BV cream (BV1, BV100, and BV500).

**Figure 3:**
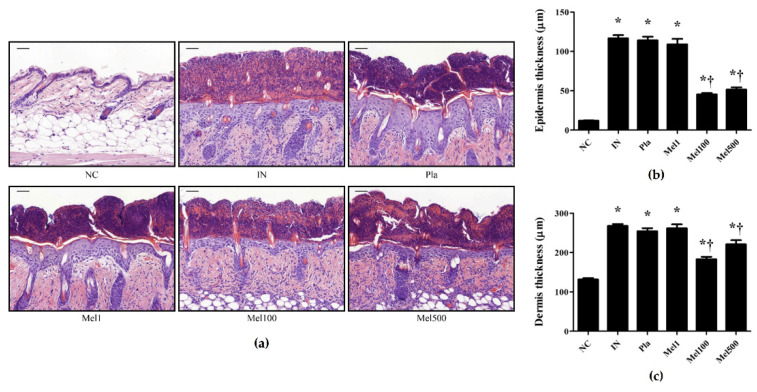
Effect of Mel in *S. pyogenes* skin infection models. At 18 h after the last topical application, biopsy specimens were taken and immediately fixed in 10% formalin, embedded in paraffin, and then stained with hematoxylin and eosin (H&E). (**a**) An increase in skin thickness and epidermal hyperplasia surrounding the infection site of *S. pyogenes* was observed by H&E staining. (**b**) Numerical value of epidermis thickness. (**c**) Numerical value of dermis thickness. The graphs are the statistical analysis for each staining. The results are expressed as mean ± SEM of three independent determinations. * *p* < 0.05 compared with the NC group; † *p* < 0.05 compared with the IN group. Magnification ×200, scale bar 50 μm. Negative control (NC); infected with *S.* *pyogenes* (IN); vaseline treatment as placebo after infection (Pla); *S. pyogenes* infection with 1, 100, and 500 μg/mL Mel cream (Mel1, Mel100, and Mel500).

After topical treatment of *S. pyogenes* infection with BV or Mel, histological analysis was performed on the dorsal skin specimens. Lesions were observed (Figure 1), beginning with a single erythematous macule that evolved into a pustule, typically leaving a honey-colored crust. In comparison with the skin of the mice in the untreated group, the severity of the lesion improved after BV treatment. A similar improvement was observed in the mice treated with Mel. Whether the infection was treated with BV or Mel, scab severity improved compared with the placebo (Pla) groups. Moreover, hematoxylin and eosin (H&E) staining of the *S. pyogenes*-infected skin revealed increased epidermal and dermal layers (Figure 2 and Figure 3). The Pla mice also showed increased thickness, whereas the BV and Mel mice showed reduced thickness. However, Masson trichrome staining did not show significant skin fibrosis in the mice infected with *S. pyogenes* (data not shown). These results, suggest that topical treatment with BV or Mel helped improve lesions induced by *S. pyogenes* skin infection.

### 2.2. BV and Mel Attenuate S. pyogenes–Induced Skin Inflammation in Mice

**Figure 4:**
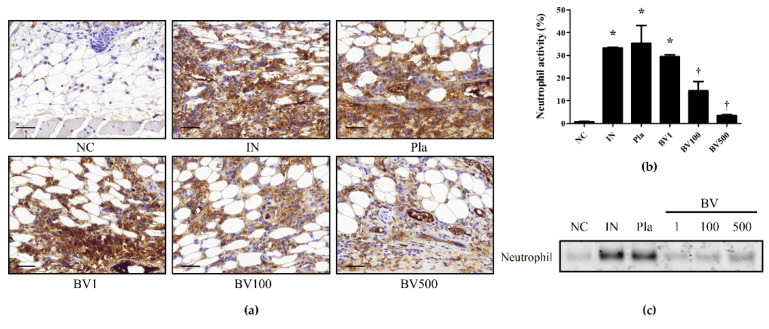
Effects of BV on neutrophil expression in *S. pyogenes* skin infection. (**a**) The representative immunohistochemical analysis images show that treatment with BV suppressed neutrophil expression in *S. pyogenes*-infected skin. (**b**) The graph shows the percentage of neutrophil-positive area. (**c**) Western-blot analysis shows the protein expressions of neutrophil in the skin tissues of each group. The results are expressed as means ± SEM. * *p* < 0.05 compared with the NC group; † *p* < 0.05 compared with the IN group. Magnification ×400, scale bar 40 μm. Negative control (NC); infected with *S. pyogenes* (IN); vaseline treatment as placebo after infection (Pla); *S. pyogenes* infection with 1, 100, and 500 μg/mL BV cream (BV1, BV100, and BV500).

**Figure 5:**
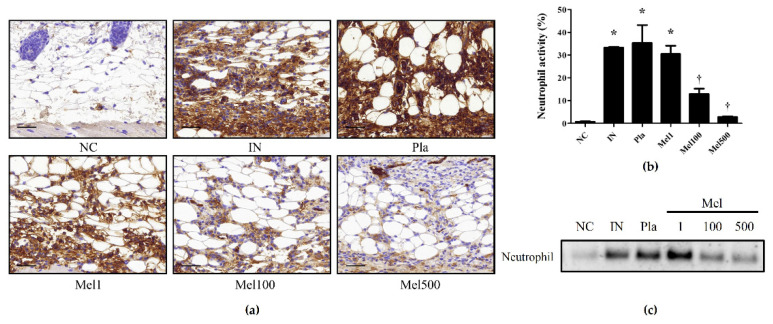
Effects of Mel on neutrophil expression in *S. pyogenes* skin infection. (**a**) The representative immunohistochemical analysis images show that treatment with Mel suppressed neutrophil expression in *S. pyogenes*-infected skin. (**b**) The graph shows the percentage of neutrophil-positive area. (**c**) Western-blot analysis shows the protein expressions of neutrophil in the skin tissues of each group. The results are expressed as means ± SEM. * *p* < 0.05 compared with the NC group; † *p* < 0.05 compared with the IN group. Magnification ×400, scale bar 40 μm. Negative control (NC); infected with *S. pyogenes* (IN); vaseline treatment as placebo after infection (Pla); *S. pyogenes* infection with 1, 100, and 500 μg/mL Mel cream (Mel1, Mel100, and Mel500).

The distribution and composition of cellular infiltrates in the skin biopsies were determined. There was a dense mix of inflammatory infiltrates composed of mononuclear cells, macrophages, mast cells, and abundant neutrophilic granulocytes. Neutrophils are the primary innate immune cells that defend against bacterial pathogens, and their presence contributes to the overall inflammatory response [27]. Immunohistochemical analysis showed that the dermal layer of *S. pyogenes*-infected skin contained more neutrophils than that of the NC mice. In contrast, low neutrophil levels were observed in the skin of BV- and Mel-treated animals, indicating the inhibition of local neutrophils recruitment (Figure 4a,b and Figure 5a,b). In line with the immunohistochemical analysis results, in Western blotting, the Pla mice showed expression levels similar to those of the IN mice, whereas the BV and Mel mice showed significantly lower expression (Figure 4c and Figure 5c). This suggests that the BV and Mel treatments could reduce the inflammatory response induced by *S. pyogenes* infection of the skin.

### 2.3. BV and Mel Inhibit Pro-Inflammatory Cytokines in S. pyogenes–Infected Mouse Skin

**Figure 6:**
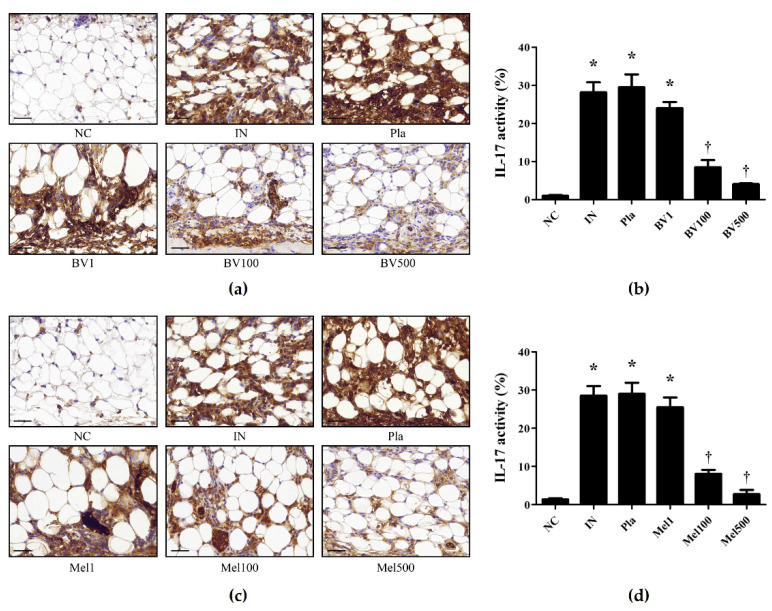
Effects of BV and Mel on the expression of IL-17A in *S. pyogenes* skin infection. (**a**,**c**) Immunochemical staining was performed to confirm the expression of IL-17A. (**b**,**d**) The graphs show the percentage of IL-17A-positive area. * *p* < 0.05 compared with the NC group; † *p* < 0.05 compared with the IN group. Magnification ×400, scale bar 40 μm. Negative control (NC); infected with *S. pyogenes* (IN); vaseline treatment as placebo after infection (Pla); *S. pyogenes* infection with 1, 100, and 500 μg/mL BV cream (BV1, BV100, and BV500); *S. pyogenes* infection with 1, 100, and 500 μg/mL Mel cream (Mel1, Mel100, and Mel500).

**Figure 7:**
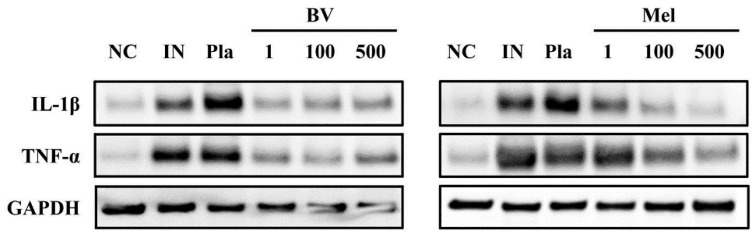
Effects of BV and Mel on the expression of pro-inflammatory cytokines. IL-1β and TNF-α were analyzed by Western-blot, and GAPDH was used to confirm equal loading of all protein samples. BV and Mel inhibit the expression of pro-inflammatory cytokines in *S. pyogenes*-infected skin tissues. Negative control (NC); infected with *S. pyogenes* (IN); vaseline treatment as placebo after infection (Pla); *S. pyogenes* infection with 1, 100, and 500 μg/mL BV cream (BV1, BV100, and BV500); *S. pyogenes* infection with 1, 100, and 500 μg/mL Mel cream (Mel1, Mel100, and Mel500).

The expression of IL-17A increased in mice infected with *S. pyogenes* and decreased in those treated with BV (Figure 6a,b) or Mel (Figure 6c,d). Similarly, Western-blot was performed to determine the effects of topical BV and Mel application on the production of inflammatory cytokines. The expression of IL-1β and TNF-α in mice infected with *S. pyogenes* significantly increased compared to the NC group, and the mice treated with a placebo showed little difference (Figure 7). Conversely, expression decreased in the BV and Mel groups, suggesting an association with the anti-inflammatory effects of BV and Mel. BV and Mel can therefore be said to inhibit the production of important inflammatory cytokines, involved in *S. pyogenes* skin infection.

### 2.4. BV and Mel Help Restore Skin Barrier Function in S. pyogenes–Infected Skin

**Figure 8:**
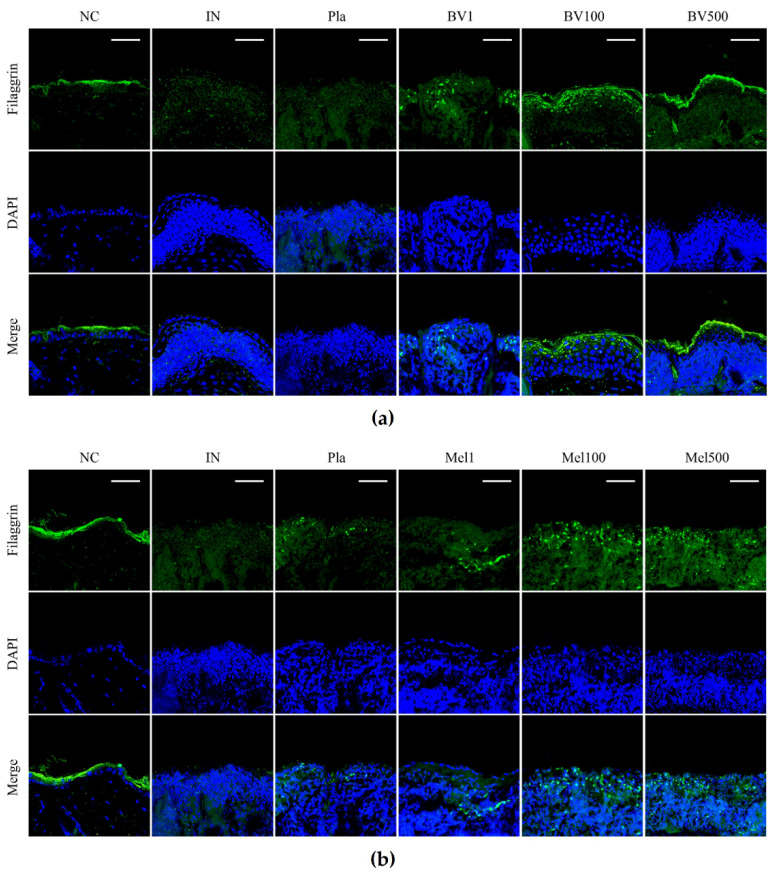
Effects of BV and Mel on filaggrin expression in *S. pyogenes*–infected mice. (**a**) Effects of BV on skin barrier function. (**b**) Effects of Mel on skin barrier function. Immunofluorescence staining for filaggrin (green), nuclei stained with DAPI (blue) and results combined (Merge). Magnification ×600, scale bar 50 μm. Negative control (NC); infected with *S. pyogenes* (IN); vaseline treatment as placebo after infection (Pla); *S. pyogenes* infection with 1, 100, and 500 μg/mL BV cream (BV1, BV100, and BV500); *S. pyogenes* infection with 1, 100, and 500 μg/mL Mel cream (Mel1, Mel100, and Mel500).

Immunofluorescent staining was performed to confirm the epidermal expression of filaggrin in the skin tissues. Filaggrin deficiency means that skin will not function properly as a barrier [28]. In mice infected with *S. pyogenes*, the expression of filaggrin was significantly decreased in comparison to the NC group, and the Pla group showed similar expression. On the other hand, filaggrin expression in the BV-treated mice showed a dose-dependent recovery pattern (Figure 8a), and Mel treatment also helped in recovery (Figure 8b). This result suggests that BV and Mel contribute to restoring skin barrier function in *S. pyogenes*-infected mouse skin.

## 3. Discussion

The skin is an organ that functions as a barrier to limit the invasion and growth of various pathogens in the body [29]. The skin protects the body from bacteria through a range of inherent defense mechanisms, but it can support the growth of bacterial pathogens under breached or moist occlusive conditions [29]. Most skin infections in the community are caused by *S. pyogenes* and *S. aureus* [30]. Infection can occur with localized clinical findings, and may indicate systemic inflammatory response syndrome when infected with a single or multiple bacterial combination [31].

Gram-positive bacteria are the most prevalent infectious agents in humans, causing a strong inflammatory response to the bacterial cell wall components and toxins secreted by some pathogenic species [32]. One such bacteria, *S. pyogenes*, releases toxins that seriously affect immune response, including peptidoglycan and lipoteichoic acid, which are cell wall components [32]. As a result of these toxic factors, superantigen activate a large proportion of the circulating T cells in the resting T cells, and the inflammatory response is further exacerbated by the cell wall components, leading to the mass production of inflammatory cytokines [32].

In traditional oriental medicine, BV is applied to the body to treat various diseases, and many studies have reported that BV, and particularly Mel, has multiple effects, including antibacterial and anti-inflammatory activities in various cell types [33]. Han et al. [34] demonstrate that BV has a sterilizing effect on *S. pyogenes*, and several other studies have shown that BV reduces the secretion of inflammatory cytokines induced by *Propionibacterium acnes* [26,35]. However, very few in vivo studies have applied BV and Mel in cases of skin disease caused by streptococcal infection, and this study, therefore, aimed to demonstrate their effects on bacterial mouse skin infection using *S. pyogenes*.

In this study, lesion severity improved with BV and Mel treatments. With similar results, an examination of the therapeutic effect of BV on acne showed a noticeable improvement in grade based on the number of inflammatory and non-inflammatory lesions as compared to the control group [36]. Moreover, our results showed a significant reduction in the thickness of both the epidermis and the dermis in the treatment groups compared with the placebo group. Likewise, Kim et al. [37] observed reduced inflammatory symptoms, such as increased thickness, edema and erythema in mice treated with BV. Similarly, An et al. [38] found that BV and Mel treatments significantly reduced dorsal skin thickness in mice with atopic dermatitis. Taken together, these findings confirm that BV and Mel have therapeutic effects on the symptoms of acute streptococcal skin infection.

Neutrophils are the main effector cells in acute inflammatory responses caused by infections [39]. IL-17A plays an important role in neutrophilic activation and induction, and produces powerful chemokines, the main chemicals attracting neutrophils [39]. These chemokines act synergistically with TNF-α, inducing and maintaining pro-inflammatory states [40]. In this study, immunochemical staining showed that IL-17A increased in skin tissues infected with *S. pyogenes* and decreased after BV and Mel treatment.

TNF-α is a multifunctional cytokine that plays an important role in the pathology of many infectious diseases through its involvement in immune regulation and inflammation [41]. IL-1 is a pro-inflammatory cytokine activated in acute inflammation. It has been shown that IL-1 is highly expressed in inflamed skin and scars [42]. IL-1β is closely related to the severity of inflammatory diseases and is beneficial for the elimination of infectious agents [43,44]. A previous study demonstrated that IL-1 signaling is significantly associated with the eradication of neutrophil-mediated *S. aureus* infection [45].

This study examined the therapeutic effects of BV and Mel on *S. pyogenes*-infected mouse skin by investigating neutrophilic expression and pro-inflammatory cytokine TNF-α and IL-1β production. The results showed that BV and Mel reduced TNF-α and IL-1β expression. These finding were confirmed by Western blotting. Similarly, a study on the therapeutic mechanism of BV in atopic dermatitis lesions caused by ovalbumin (a major egg white protein) found that BV inhibited pro-inflammatory cytokines, such as TNF-α [46]. Another study documented the protective effects of Mel on the inflammatory response to *P. acnes* [25]. The results of our study confirm that BV and Mel suppress inflammation, demonstrating their effectiveness in treating skin lesions caused by *S. pyogenes*.

Filaggrin, particularly important for the formation of the skin barrier, plays a fundamental role in terminal epidermal differentiation and affects a range of general dermatological diseases [47]. Kim et al. [37] found that ovalbumin-induced atopic dermatitis can be improved by decreasing filaggrin levels, while An et al. [38] demonstrate that the topical application of BV or Mel restores abnormal epidermal differentiation by recovering filaggrin expression. As with these previous reports, filaggrin expression was found to be decreased in the streptococcal-infected skin tissue of this study and to recover toward higher concentrations in the groups treated with BV or Mel. These results show that topical treatment of BV or Mel can restore the skin’s barrier function when damaged by streptococcal infection. Consequently, BV or Mel can be said to contribute to the normalization of damaged barrier function, prevent the occurrence of secondary infection, and avoid deterioration of the lesion. Taken together, these results confirm that BV and Mel have therapeutic effects on acute streptococcal skin infection symptoms.

The safety of long-term topical BV and Mel treatments remains uncertain. In this study, although lesion severity tended to decrease, differences between individuals were observed. Furthermore, complete recovery was not observed, as this would have required a longer experimental period than was available. Further investigation is therefore needed to determine whether long-term BV or Mel treatment can completely heal infection sites and what side effects it may have.

## 4. Conclusions

This study investigated the potential of topical BV and Mel treatments for *S. pyogenes* skin infection. A tape-stripped mouse infection model was used to induce superficial skin infection with *S. pyogenes*. This results show that short-term BV and Mel treatments can promote wound healing in the superficial skin layer. This study provides further evidence of the potential therapeutic effects of BV and Mel on skin infection caused by *S. pyogenes*, suggesting that with further optimization such as adjusting the application period or concentrations, they could be used to develop novel treatments for streptococcal skin infections.

## 5. Materials and Methods

### 5.1. Bacterial Strains and Culture Conditions

The *S. pyogenes* strain (KCCM 11875) was grown aerobically overnight on blood agar with 5% sheep blood plates at 37 °C. The bacterial cells were harvested and resuspended in phosphate buffered saline then centrifuged at 4000 rpm for 10 min. The process was repeated three times. The washed bacteria were resuspended in distilled water for use in the following experiments. *S. pyogenes* were adjusted to 1.0 × 10^8^ colony-forming units (CFU)/mL at an absorbance wavelength of 580 nm.

### 5.2. Animals

Six-week-old female Balb/C mice were purchased (Samtako Inc., Osan, Korea) and housed at 55% humidity and 22 ± 2 °C in a 12-h light-dark cycle and allowed food and water ad libitum for 7 days. Animal care and all experimental procedures were approved and conducted in accordance with the guidelines of the Institutional Animal Care and Use Committee of the Catholic University of Daegu (Approval number: DCIAFCR-210607–08-Y; Approved date: 7 June 2021).

### 5.3. Mouse Skin Infection Model

The mice were randomly divided into nine equal groups (*n* = 6 per group): negative control (NC); *S. pyogenes* infection without treatment (IN); *S. pyogenes* infection with Vaseline as placebo (Pla); *S. pyogenes* infection with 1, 100, 500 μg/mL BV (Dongsung Bio Pharm, Korea; BV1, BV100, and BV500); and *S. pyogenes* infection with 1, 100, 500 μg/mL Mel (Chungjin Biotech, Ansan, Korea; Mel1, Mel100, and Mel500). The Pla group was topically treated only with vaseline as a placebo. The BV and Mel groups were topically treated with BV and Mel, mixed with vaseline (0.5 g per mouse). The treatment was initiated 48 h post-infection and performed once more 6 h after first treatment. The fur on the back of all mice was shaved, and the animals were put under brief isoflurane anesthesia. The dorsal skin was stripped with autoclave tape. In order to standardize the degree of barrier disruption elicited by the tape stripping, transepidermal water loss (TEWL) was measured using a barrier device (gpower, Seoul, South Korea). Each mouse was stripped between 7 and 10 times until TEWL reached approximately 70 g/m^2^h. Then, living *S. pyogenes* (1 × 10^8^ CFU/50 μL in distilled water) were topically applied and well spread out. At 18 h after the last topical treatment, the mice were sacrificed and the superficial wound specimens excised for dermatohistopathology.

### 5.4. Histological Analysis

The dorsal skin specimens were fixed in 10% formalin at room temperature (RT) and subsequently dissected, dehydrated, and embedded in paraffin. The paraffin-embedded tissues were cut into 4 µm sections which were then mounted on glass slides and stained with hematoxylin and eosin (H&E) according to standard protocols.

### 5.5. Immunohistochemical Analysis

The tissue sections were deparaffinized with xylene and dehydrated in gradually decreasing concentrations of ethanol. The sections were incubated with a primary antibody (1:100) for 1 h at 37 °C. The signal was visualized using the EnVision System (Dako, Carpinteria, CA, USA) for 30 min at 37 °C; the coloring reagent was 3,3′-diaminobenzidine tetrahydrochloride, and hematoxylin was used as the counterstain. The primary antibodies were anti-neutrophil and anti-IL-17A (Abcam, Cambridge, UK). All slides were scanned using a Pannoramic^®^ MIDI slide scanner (3DHISTECH, Budapest, Hungary).

### 5.6. Immunofluorescence Analysis

The dorsal skin specimens were deparaffinized with xylene and dehydrated in gradually decreasing concentrations of ethanol. The primary antibody (1:200) was added followed by incubation for 2 h at 37 °C, and secondary antibody (1:200) was then performed for 1 h at RT. The nuclei were labeled with 4′, 6-diamidino-2-phenylindole (DAPI; 1:1000) at RT for 2 min. The antibodies were anti-filaggrin (Enzo Life Sciences, Lausen, Switzerland) and anti-rabbit-biotinylated secondary antibodies conjugated with fluorescein isothiocyanate (Invitrogen; Thermo Fisher Scientific, Inc., Waltham, MA, USA). The slides were mounted using a fluorescence mounting medium (Dako; Agilent Technologies, Inc., Santa Clara, CA, USA), and the stained slides were imaged using a NIKON^®^ A1+ confocal microscope (Nikon, Tokyo, Japan).

### 5.7. Western Blot Analysis

Protein samples were extracted from the skin specimens using Cell Lytic™ M protein extraction solution (Sigma-Aldrich, St. Louis, MO, USA), according to the manufacturer’s recommendations. After incubation for 30 min on ice, the total extract was centrifuged at 12,000 rpm at 4 °C for 10 min and the supernatant was collected. The protein concentration was measured with the Bradford assay (Bio-Rad Laboratories, Hercules, CA, USA) at 595 nm using a spectrophotometer.

The protein samples were separated using precast gradient polyacrylamide gels (Bolt^TM^ 4–12% Bis-Tris Plus Gels; Thermo Fisher Scientific, Waltham, MA, USA) and transferred to a nitrocellulose membrane (GE Healthcare, Chicago, IL, USA). The membrane was blocked in 5% bovine serum albumin for 1 h at RT and then incubated overnight with the primary antibody at 4 °C. The membranes were then incubated with horseradish peroxidase-conjugated secondary antibodies for 1 h at RT. The signals were detected using enhanced chemiluminescence detection reagents (Thermo Fisher Scientific, Waltham, MA, USA). Signal intensity was analyzed using the iBright™ CL1500 Imaging System (Thermo Fisher Scientific, Waltham, MA, USA) and quantified using the Image Lab Software (Bio-Rad Laboratories, Hercules, CA, USA). The protein expression levels were normalized to GAPDH (Cell Signaling, Beverly, MA, USA) expression values. The primary antibodies used were anti-interleukin-1 beta (IL-1β; Santa Cruz Biotechnology, Santa Cruz, CA, USA), anti-tumor necrosis factor-alpha (TNF)-α, anti-neutrophil (Abcam, Cambridge, UK), and anti-GAPDH (Cell Signaling, Beverly, MA, USA).

### 5.8. Statistical Analysis

All results were presented as mean ± standard error of the mean (SEM) and statistical significance was tested using Prism 5 (GraphPad Software, Inc., San Diego, CA, USA). Statistical significance was tested by one-way analysis of variance with a Tukey’s multiple comparison test and difference with *p* < 0.05 were considered significant.

## Data Availability

Not applicable.
